# Umbilical Cord Blood-Derived Cells Can Reconstruct Hematopoiesis in an Aplastic Anemia Animal Model

**DOI:** 10.1155/2024/4095268

**Published:** 2024-08-12

**Authors:** Zesong Chen, Chen Yang, Jiang Ji, Miao Chen, Bing Han

**Affiliations:** ^1^ Department of Hematology Peking Union Medical College Hospital Chinese Academy of Medical Science and Peking Union Medical College, Beijing 100730, China; ^2^ Department of Oncology Cancer Hospital Chinese Academy of Medical Sciences Shenzhen Hospital, Shenzhen, China

## Abstract

**Objectives:**

To explore the efficacy and the mechanism of the umbilical cord-derived cells combined with cyclosporine A (CsA) in treating aplastic anemia (AA) in mice.

**Methods:**

Immune-mediated AA model mice were treated with CsA + UC mesenchymal stem cells (UC-MSC), CsA + umbilical cord blood regulatory T cells (UCB-T_reg_), UC-MSC, UCB-T_reg_, CsA alone, or blank control, respectively (*n* = 9 mice/group). CsA and the cell infusion was administered on d0. Routine peripheral blood testing was performed once weekly; bone marrow colony culture, bone marrow cell flow cytometry, peripheral blood T cell subsets, and serum inflammatory cytokines tests were performed on d14. Transcriptome sequencing was performed for cells from CsA + UC-MSC, CsA + UCB-T_reg_, and CsA groups to detect the possible related genes. Gene function cluster and signal pathway enrichment analysis were also performed.

**Results:**

Blank control mice died due to pancytopenia within 21 days, whereas mice in other groups survived for >28 days. On d14, the CsA + UC-MSC and CsA + UCB-T_reg_ groups had higher white blood cell (WBC) counts than the other groups (*p* < 0.05), along with higher burst-forming unit (BFU) and colony-forming unit-granulocyte, macrophage (CFU-GM) counts (*p* < 0.01). The CsA + UC-MSC group had the highest BFU count (*p* < 0.01). The CsA + UC-MSC and CsA + UCB-T_reg_ groups exhibited the highest bone marrow CD34^+^ cell proportion (9.68% ± 1.35% and 8.17% ± 0.53%, respectively; *p* < 0.01). Tumor necrosis factor (TNF)-*α* and interleukin (IL)-2 levels in the CsA + UC-MSC group (*p* < 0.05) and TNF-*α*, interleukin-2, and interferon (INF)-*γ* levels in the CsA + UC-T_reg_ group (*p* < 0.01) were lower than those in the CsA group. Compared with CsA treatment, CsA + UC-MSC significantly downregulated the histone methylation pathway (*p* < 0.05), whereas CsA + UCB-T_reg_ significantly upregulated energy metabolism processes (*p* < 0.05). Treatment with CsA + UC-MSC upregulated superoxide dismutase activity compared with CsA + UCB-T_reg_ treatment.

**Conclusions:**

Adding UC-MSC or UCB-T_reg_ to CsA markedly enhanced the reconstruction of hematopoiesis in AA mice, with UC-MSC eliciting greater efficiency than UCB-T_reg_. Accordingly, the addition of these cells could further improve immune abnormalities.

## 1. Introduction

Aplastic anemia (AA) is characterized by low bone marrow (BM) hyperplasia and pancytopenia. Acquired AA can be further divided into secondary and idiopathic AA based on the presence of clear secondary factors [1]. Abnormalities in hematopoietic stem cells, hematopoietic microenvironment, and immune system have been observed in idiopathic AA [2, 3, 4]. BM-derived mesenchymal stem cells (MSCs) are well known to participate in hematopoietic support and immune regulation, both of which are reduced in patients with idiopathic AA [5]. Likewise, patients with AA reportedly exhibit a reduced number of regulatory T cells (T_regs_), although functional defects in these cells were enhanced [6, 7]. Immunosuppressive therapy (IST) is reportedly effective in 70%–80% of patients with AA who are ineligible to undergo transplantation, although novel treatment options need to be explored for patients who fail to benefit from IST [1]. Preclinical and clinical studies have confirmed the safety and efficacy of infusion therapy with MSCs or T_regs_ derived from the BM or other tissues in patients with AA [8, 9, 10, 11, 12].

Umbilical cord blood (UCB) is typically collected from the umbilical cord (UC) and placenta of newborns. Owing to the abundance of hematopoietic stem cells and immature immune cells, UCB can be employed in transplantation and transfusion therapy for hematological malignancies and BM failure diseases [13, 14, 15, 16]. In patients with AA, cord blood infusion therapy can hasten the onset of immunosuppressive therapy and improve the long-term prognosis [14]. Importantly, there have been considerable advancements in technologies applied to isolate and culture human UC-derived MSCs (UC-MSC) and human UCB-T_reg_ from fresh UC and UCB [17, 18]. UC-MSCs exhibit stronger hematopoietic support and immune regulation function than BM-derived MSCs, and UC-MSC infusion may elicit robust efficacy, while UCB-T_reg_ may be more suitable for infusion therapy [19, 20, 21]. MSCs were found to induce CD4^+^ T cells to enhance CD25 and forkhead box protein P3 (Foxp3) expression under coculture conditions *in vitro*. UC-MSC infusion may play a therapeutic role by predominantly increasing the number of T_reg_ in patients with autoimmune diseases [19, 22]. Although the application of UC-MSC therapy in patients with AA has been reported, to the best of our knowledge, investigations on treatment with UCB-T_reg_ or comparisons between UC-MSCs and UCB-T_reg_ are lacking.

In the current study, in the context of cyclosporine A (CsA) therapy, we examined the effects of UC-MSC and UCB-T_reg_ infusion in an immune-mediated AA mouse model. We attempted to illustrate the differences between UC-MSC and UCB-T_reg_ infusions in terms of their ability to induce hematopoietic stimulation, immune-regulation effects, and gene transcription to lay a foundation for further advancing cell infusion therapy.

## 2. Materials and Methods

### 2.1. Induction of AA Murine Model and Umbilical Cell Preparation

A murine AA model was established as described previously [9, 23, 24]. Lymph node cells from C57BL/6 (male, 49–72 days old, SPF grade) donors were homogenized, washed, and filtered. In total, 54 C57BL/6 mice (male, 49–72 days old, SPF grade) were subjected to sublethal irradiation (5 Gy Co-60 total body irradiation) followed by the administration of a single dose of 5 × 10^6^ lymph node cell infusion through the lateral tail vein within 4 hr postirradiation. The AA model mice were randomly assigned to six groups and administered different treatments, as shown in Table 1. Human UC-MSCs and human UCB-T_reg_ were provided by Shandong Cord Blood Bank, China. UC-MSC or UCB-T_reg_ infusion was administered within 24 hr after AA model induction. Table 1 presents the therapeutic drugs and cells used in the study. All experimental operations on mice were conducted in accordance with the Declaration of Helsinki and were approved by the ethics committee of Peking Union Medical College Hospital, Beijing, China.

### 2.2. Survival Status and Routine Blood Monitoring

The survival status of mice in each group was observed daily, starting from d0. Routine blood tests were conducted on days 0, 7, 14, and 28 by collecting blood from the intraorbital venous plexus of the mice.

### 2.3. Flow Cytometry

Peripheral blood was collected from the intraorbital venous plexus, and BM cells were extracted from the dissected tibia and femur of each mouse. The BM cell suspension was repeatedly pipetted to disperse cells and then filtered through a 200-mesh screen. The suspension was centrifuged at 500 × *g* for 5 min at room temperature, and the cells were resuspended in phosphate-buffered saline for counting. After processing with red blood cell lysate, peripheral blood lymphocytes and BM cells were centrifuged at 4°C. Monoclonal antibodies were added and incubated according to the instructions provided with the antibodies. Monoclonal antibodies against murine CD4-FITC, CD8-perCP-Cy5.5, CD25-APC, Foxp3-PE, and CD34-APC were procured from BD Biosciences. Stained and unstained cells as blank control were analyzed using a FACSC auto II flow cytometer (BD Biosciences).

### 2.4. BM Colony Assay

BM cells were collected and filtered as previously described and plated at a density of 2.0 × 10^4^/plate in methylcellulose-based medium with recombinant cytokines (including erythropoietin (EPO)) for mouse cells (MethoCult™ GF M03434, STEMCELL Technology Inc., Canada). The colonies were maintained at 37°C in a 5% CO_2_ incubator for 12 days. The number of BM colonies was counted using an inverted microscope.

### 2.5. Enzyme-Linked Immunosorbent Assay (ELISA) Detection of Serum Cytokines

The venous blood samples were centrifuged at room temperature for 15 min at 4,000 rpm, and the serum (supernatant) was collected for ELISA detection. The expression of inflammatory factors, including interferon (INF)-*γ*, tumor necrosis factor (TNF)-*α*, and interleukin (IL)-2, were detected using ELISA kits (R&D Systems) according to the manufacturer's instructions. In brief, 50 *μ*L of detection diluent + 50 *μ*L of sample, standard, or blank control were added to each well, and the optical density (OD) at a wavelength of 450 nm was measured after incubation. The concentration of each sample was determined by inserting the OD values of each sample well into the standard curve.

### 2.6. Transcriptome Sequencing

Total RNA from the peripheral blood sample of mice was extracted using TRlzol reagent (Life Technologies, California, USA) according to the manufacturer's instructions. RNA concentration and purity were measured using NanoDrop 2000 (Thermo Fisher Scientific, Wilmington, DE, USA). RNA integrity was assessed using the RNA Nano 6000 assay kit on the Agilent Bioanalyzer 2100 system (Agilent Technologies, CA, USA). Library preparation and transcriptome sequencing were generated using the Hieff NGS Ultima Dual-mode mRNA Library Prep kit for Illumina (Yeasen Biotechnology (Shanghai) Co., Ltd.) and the Illumina NovaSeq platform. After mapping to the reference genome and novel transcripts prediction, gene function was annotated based on the following databases: Nr, NCBI nonredundant protein sequences; KOG/COG, Clusters of Orthologous Groups of proteins; P_fam_, Protein family; KO, KEGG Ortholog database; and GO, Gene Ontology. Differential expression genes were screened with fold-change ≥1.5 and *p*  < 0.05, and differential genes were subjected to functional annotation and enrichment analysis.

### 2.7. Data Analysis

Blood and BM cell composition data were analyzed using IBM SPSS 25.0 software (IBM; Armonk, NY, USA) and Flowjo V10 software (Tree Star, Inc., Ashland, OR, USA). Analysis of variance (ANOVA), *t*-test, and Mann–Whitney U nonparametric test were used to evaluate differences in data. Statistical significance is indicated using *p*  < 0.05, *p*  < 0.01, and *p*  < 0.001 levels ( ^*∗*^,  ^*∗∗*^, and  ^*∗∗∗*^, respectively).

## 3. Results

### 3.1. Combination Therapy Enhanced the Recovery of Peripheral Blood Cell Count

**Figure 1:**
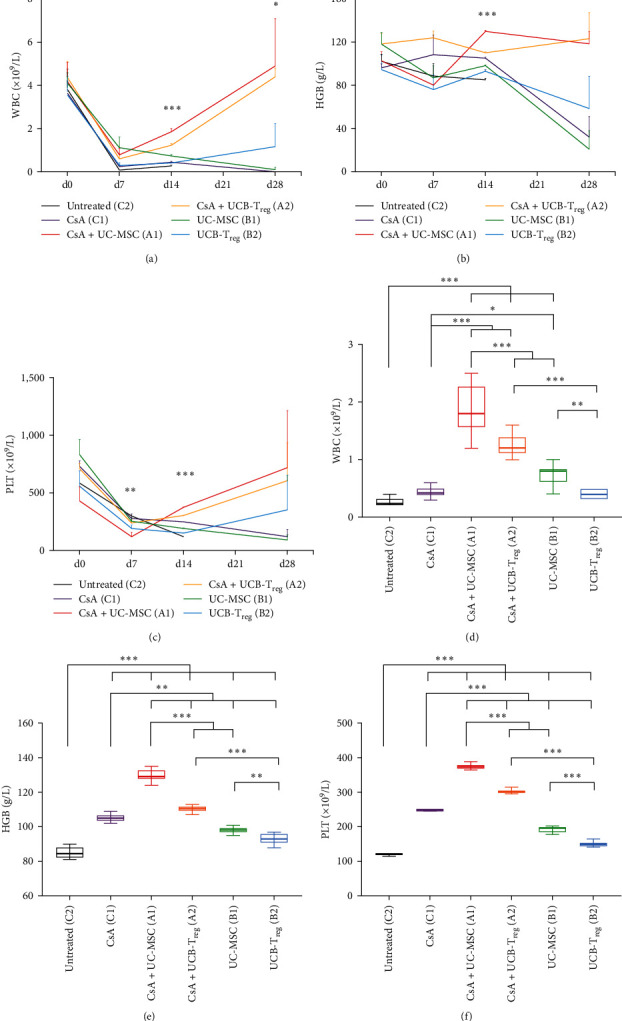
Induction of AA murine model and hematopoiesis recovery in mice with different treatments. (a–c) White blood cell (WBC), hemoglobin (HGB), and platelet (PLT) levels in mice subjected to different treatments at different time points (represented as the means and SD).  ^*∗*^,  ^*∗∗*^, and  ^*∗∗∗*^ indicate a significant difference among all groups (*p* < 0.05, *p* < 0.01, and *p* < 0.001, respectively). (d–f) WBC, HGB, and PLT levels in mice subjected to different treatments at D14 (represented as means ± SD).  ^*∗*^,  ^*∗∗*^, and  ^*∗∗∗*^ indicate a significant difference between two groups (*p* < 0.05, *p* < 0.01, and *p* < 0.001, respectively).

Based on the preliminary results, untreated mice (group C2) revealed a significant simultaneous decline in peripheral blood ternary sorts on d7 and d14, and all untreated mice died within 21 days postradiation. As shown in Figures 1(a), 1(b), and 1(c), the number of blood cells in all mice was the lowest on d7 after model generation, gradually recovering thereafter. Considering each group, the white blood cell (WBC) count did not differ at d0 and d7 but differed significantly at d14 and d28 (d14, *p*  < 0.001 and d28, *p*  < 0.05). The hemoglobin level differed significantly between groups only on d14 (*p*  < 0.001) but not on d0, d7, and d28. There were no differences in platelet levels in each group at d0, although a significant difference was observed at d7 and d14 (d7, *p*  < 0.01 and d28, *p*  < 0.001), and the difference disappeared at d28.

There were no significant differences in baseline blood cell counts in the above groups. On d14, the CsA + UC-MSC group exhibited significantly better recovery in all three blood cells than the UC-MSC, CsA, and blank control groups (all *p*  < 0.001). Likewise, the CsA + UCB-T_reg_ group had significantly better hematopoiesis recovery than the UCB-T_reg_, CsA, and blank control group (Figures 1(d), 1(e), and 1(f)). On d28, there were no significant differences in blood cell counts between the different treatment groups; however, the CsA + UC-MSC and CsA + UCB-T_reg_ groups exhibited higher WBC counts than the other groups (*p*  < 0.05). Accordingly, the UC-MSC infusion therapy was superior to the UCB-T_reg_ infusion therapy in terms of the speed of recovery of the tertiary blood system with or without CsA treatment (Figures 1(a), 1(b), 1(c), 1(d), 1(e), and 1(f)).

### 3.2. Combination Therapy Elicited Superior Recovery of the BM Colony

**Figure 2:**
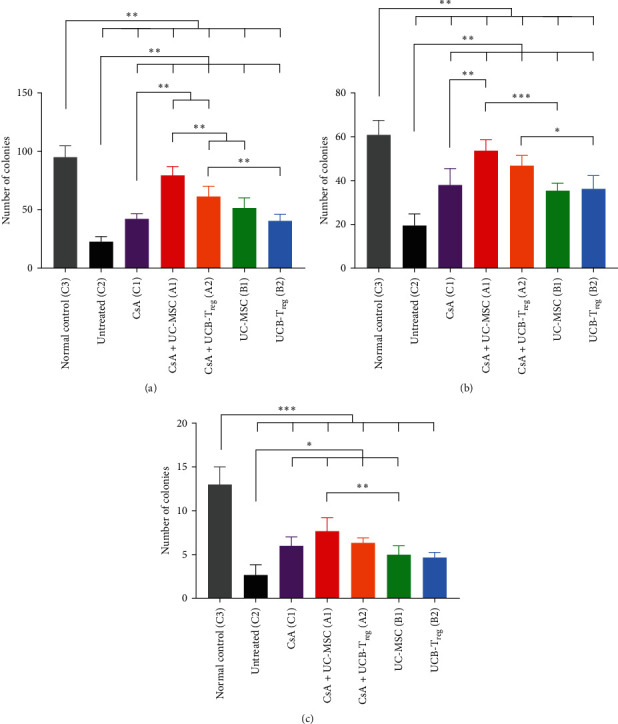
Colony numbers in different groups (*n* = 3; on 2 × 10^4^ bone marrow nucleated cells). (a) Burst-forming unit (BFU) colony numbers in different groups. (b) Colony-forming unit-granulocyte, macrophage (CFU-GM) colony numbers in different groups. (c) Colony-forming unit-granulocyte, erythroid, macrophage, megakaryocyte (CFU-GEMM) colony numbers in different groups. Data are presented as means ± standard deviation (SD).  ^*∗*^,  ^*∗∗*^, and  ^*∗∗∗*^ indicate a significant difference between two groups (*p*  < 0.05, *p*  < 0.01, and *p*  < 0.001, respectively). CsA, cyclosporine A; MSC, mesenchymal stem cells; UC, umbilical cord; UCB, umbilical cord blood; and T_reg_, regulatory T cells.

Untreated mice (group C2) showed a significant decline in the BM nucleated cells (BMNC) colony cultures at d14 (*p*  < 0.01). All treated groups had significantly improved BM colony counts than the control group (*p*  < 0.01), as shown in Figure 2. The CsA + UC-MSC and CsA + UCB-T_reg_ groups had higher burst-forming unit (BFU) and colony-forming unit-granulocyte, macrophage (CFU-GM) colony counts than the CsA, UC-MSC, or UCB-T_reg_ groups (*p*  < 0.01); the CsA + UC-MSC group exhibited a better recovery in the BFU colony count than the CsA + UCB-T_reg_ group (*p*  < 0.01). CFU-GM and colony-forming unit-granulocyte, erythroid, macrophage, megakaryocyte (CFU-GEMM) numbers were higher in the CsA + UC-MSC than those in the CsA + UCB-T_reg_ group, although the difference was not significant.

### 3.3. Combination Therapy Enhanced the Numbers of BM CD34^+^ Cells

**Figure 3:**
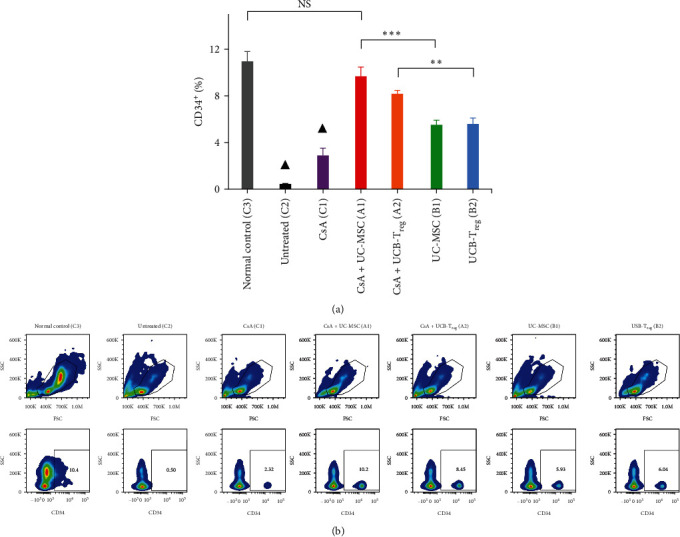
CD34^+^ bone marrow nucleated cells analysis in different treatment groups. (a) CD34^+^cell population ratio of mice in each treatment group. (b) Detection of bone marrow CD34^+^cell by flow cytometry.  ^*∗*^,  ^*∗∗*^, and  ^*∗∗∗*^ indicate a significant difference between two groups (*p*  < 0.05, *p*  < 0.01, and *p*  < 0.001, respectively). NS indicates no significant difference between the two groups. ▲ indicates a significant difference between this group and any other group (all meet the requirements of *p*  < 0.01). CsA, cyclosporine A; MSC, mesenchymal stem cells; UC, umbilical cord; UCB, umbilical cord blood; and T_reg_, regulatory T cells.

On d14, the proportion of CD34^+^ cells in the BM significantly decreased in blank control mice when compared with those in normal mice (*p*  < 0.01). Conversely, all treated mice had significantly increased proportions of BM CD34^+^ cells (*p*  < 0.01) when compared with the blank control, as shown in Figure 3. Combined treatment groups, that is, the CsA + UC-MSC and CsA + UCB-T_reg_ groups, had a higher number of CD34^+^ cells than the CsA monotherapy group (CsA + UC-MSC vs. CsA, 9.68% ± 1.35% vs. 2.89% ± 0.60%, *p*  < 0.01 and CsA + UCB-T_reg_ vs. CsA, 8.17% ± 0.53% vs 2.89% ± 0.60%, *p*  < 0.01). No significant difference was detected between the CsA + UC-MSC and CsA + UCB-T_reg_ groups (*p*= 0.073), and the CsA + UC-MSC group exhibited similar findings to the normal group in terms of CD34^+^ BMNC analysis (*p*=0.121).

### 3.4. Combined Cell and Drug Therapies Restored the T Cell Balance in AA Mice

**Figure 4:**
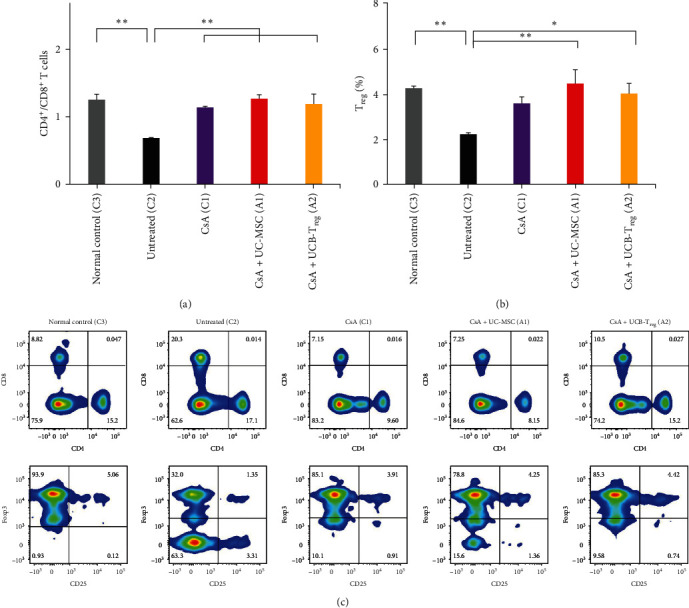
Changes in T cell subsets in different treatment groups. (a) The radio of CD4^+^/CD8^+^ in each treatment group. (b) The ratio of T_reg_/CD4^+^ in each treatment group. (c) Detection of CD4^+^/CD8^+^ T cell subsets and T_reg_ (CD25^+^ and Foxp3^+^) subsets in CD4^+^ T cell subsets in different treatment groups by flow cytometry.  ^*∗*^,  ^*∗∗*^, and  ^*∗∗∗*^ indicate a significant difference between two groups (*p*  < 0.05, *p*  < 0.01, and *p*  < 0.001, respectively). CsA, cyclosporine A; MSC, mesenchymal stem cells; UC, umbilical cord; UCB, umbilical cord blood; and T_reg_, regulatory T cells.

T cell subsets were detected in normal mice and untreated, CsA + UC-MSC, CsA + UCB-T_reg_, and CsA single treatment groups. As shown in Figures 4(a), 4(b), and 4(c), compared with normal mice, AA mice showed extensive infiltration of CD8^+^ T cells in peripheral blood, and the proportion of CD4^+^/CD8^+^ T cells and that of T_reg_/CD4^+^ T cells rapidly decreased. Changes in T cell subsets were consistent with the immunological features observed in human AA. Following the administration of various treatments, the ratio of CD4^+^/CD8^+^ T cells was significantly increased (CsA + UC-MSC group, 1.27 ± 0.05; CsA + UCB-T_reg_ group, 1.18 ± 0.14; CsA group, 1.14 ± 0.02; and untreated group, 0.68 ± 0.01; *p*  < 0.05). Moreover, the T_reg_ radio was significantly increased after treatment (CsA + UC-MSC group, 4.47 ± 0.63; CsA + UCB-T_reg_ group, 4.04 ± 0.46; CsA group, 3.59 ± 0.28; and untreated group, 2.24 ± 0.04; *p*  < 0.05). There were no significant differences in CD4^+^/CD8^+^ T cells or the T_reg_ radio among the CsA + UC-MSC, CsA + UCB-T_reg_, CsA single drug groups, and the normal control group (*p*  > 0.05; Figure 4(c)).

### 3.5. Combination Therapy Reduced Serum Inflammatory Markers in AA Mice

**Figure 5:**
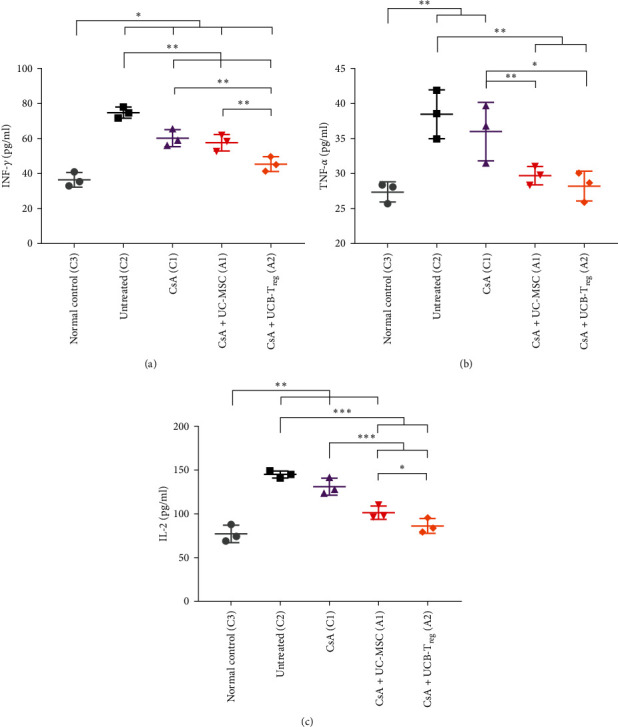
Serum levels of inflammatory factors in different treatment groups. (a) INF-*γ* levels in different treatment groups. (b) TNF-*α* levels in different treatment groups. (c) IL-2 levels in different treatment groups.  ^*∗*^,  ^*∗∗*^, and  ^*∗∗∗*^ indicate a significant difference between the two groups (*p* < 0.05, *p*  < 0.01, and *p*  < 0.001, respectively). CsA, cyclosporine A; MSC, mesenchymal stem cells; UC, umbilical cord; UCB, umbilical cord blood; T_reg_, regulatory T cells; IFN-*γ*, interferon-*γ*; IL-2, interleukin-2; and TNF-*α*, tumor necrosis factor-*α*.

Serum levels of inflammatory factors (INF-*γ*, TNF-*α*, and IL-2) were detected by ELISA on d14, as shown in Figure 5. Compared with the levels in normal mice, the levels of all inflammatory factors were significantly elevated in untreated mice, which is consistent with the disease state (*p*  < 0.05). The levels of INF-*γ*, TNF-*α*, and IL-2 were reduced in the CsA + UC-MSC and CsA + UCB-T_reg_ groups when compared with those in the untreated group (all *p*  < 0.05). Treatment with CsA reduced levels of TNF-*α* and IL-2 (all *p*  < 0.05), whereas those of INF-*γ* showed no significant difference (*p*  > 0.05). The levels of INF-*γ* and IL-2 were further reduced in the CsA + UCB-T_reg_ group when compared with those in the CsA + UC-MSC group (*p*  < 0.05). Following the CsA + UCB-T_reg_ treatment, TNF-*α* and IL-2 reached levels comparable with those in the normal control group, showing no significant differences. Similarly, after CsA + UC-MSC treatment, TNF-*α* levels aligned with those of the normal control group, exhibiting no notable variance (all *p*  > 0.05).

### 3.6. Transcriptome Analysis of Mechanisms Underlying Enhanced Hematopoiesis following Combination Treatment

**Figure 6:**
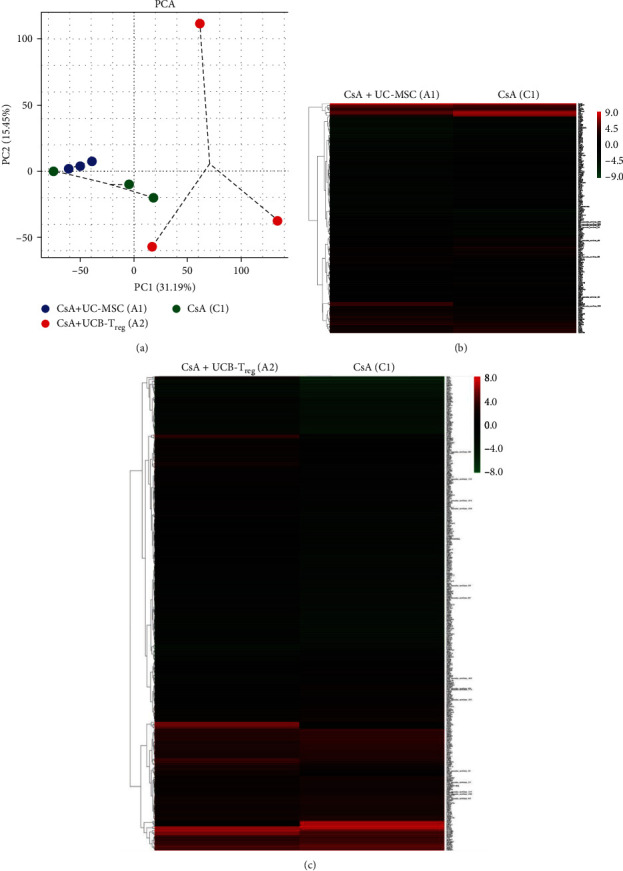
PCA plot and heatmap of significantly altered genes between two kinds of infusion treatments and CsA monotherapy. (a) PCA plot of all the groups. (b) Heatmap of significantly altered genes between the CsA + UC-MSC and CsA group. (c) Heatmap of significantly altered genes between the CsA + UCB-T_reg_ and CsA group. CsA, cyclosporine A; MSC, mesenchymal stem cells; UC, umbilical cord; UCB, umbilical cord blood; T_reg_, regulatory T cells; and PCA, principal component analysis.

Given that the treatment with CsA + UC-MSC and CsA + UCB-T_reg_ exhibited better hematopoiesis reconstruction ability than CsA monotherapy, we next aimed to explore the possible mechanism. Accordingly, peripheral blood was collected from mice in the CsA + UC-MSC, CsA + UCB-T_reg_, and CsA groups for transcriptome sequencing. Compared with the CsA group, the CsA + UC-MSC group had 28 genes significantly up- and 113 genes downregulated, and the difference was mainly focused on the downregulation of genes related to histone methylation, including Kmt2a, Kmt2c, Kdm4a, and Arid4a; in the CsA + UCB-T_reg_ group, we identified 252 and 87 upregulated and downregulated genes, respectively, with the difference mainly observed in the upregulation of energy metabolism, including tricarboxylic acid cycle, oxidative phosphorylation and glycolytic process (Figure 6). Compared with the CsA + UCB-T_reg_ group, the CsA + UC-MSC group exhibited 420 and 407 upregulated and downregulated genes, respectively, with the difference primarily focused on the upregulation of superoxide dismutase (SOD) activity and oxidative phosphorylation pathway in the CsA + UC-MSC group.

## 4. Discussion

The pathogenesis of idiopathic AA has long been summarized by a “seeds, soil, worms” theory [4]. Recently, BM-MSCs and T_reg_ in patients with AA were shown to exhibit reduced number and loss of function [6, 7]. BM-MSCs are an important constituent of the BM hematopoietic microenvironment, supporting hematopoiesis and functioning as an immune regulator by interacting with immune cells or secreting inflammatory cytokines [25]. T_reg_ cells are a group of T lymphocytes characterized by the coexpression of CD4, CD25, and Foxp3, capable of inhibiting the proliferation, activation, and killing of effector T cells by directly contacting or secreting cytokines. Impaired T_reg_ function has been reported in patients with AA, which could not be attributed to the attack by effector T cells [7].

Owing to the close relationship between AA pathogenesis and the dysfunction of MSC and T_reg_, the application of MSC or T_reg_ cells to treat AA has been examined in a few reports, either in humans or in animal models, eliciting promising results [5, 8, 25, 26, 27]. Cord blood UC-MSC and UCB-T_reg_ were easier to obtain and exerted stronger immune regulation ability than MSC or T_reg_ cells derived from other tissues. T_reg_ cells play the most crucial role in immune regulation and may be the key component for cord blood transfusion. However, the separation of T_reg_ can be complicated and unstable when compared with MSC separation; therefore, it is important to identify which cell is more efficient *in vivo*, as well as the possible mechanism [19, 20, 21]. However, only a few studies have focused on UC-MSC infusion therapy for patients with AA or in AA animal models. To date, no study has explored the potential of UCB-T_reg_ infusion for treating AA [28].

In the current study, we further compared the effects of UC-MSC or UCB-T_reg_ addition to CsA in our AA mouse model. To the best of our knowledge, this is the first study to apply UCB-T_reg_ infusion and CsA + UCB-T_reg_ therapy to treat AA model mice. Furthermore, this study is the first to comprehensively compare the efficacy of UCB-T_reg_ therapy with UC-MSC therapy in AA model mice, with or without an immunosuppressant.

Our AA model mimicked patients with AA—not only did mice exhibit BM failure, but showed abnormal immunity, as observed in patients with AA. These defects can be partly corrected with CsA alone, as reported previously [29]. Likewise, rescue with UC-MSC or UCB-T_reg_ alone was also found to be efficient, as evidenced by the avoidance of early deaths and restoration of hematopoiesis, which was similar to CsA monotherapy. Next, we added UC-MSC or UCB-T_reg_ to CsA, which elicited better recovery in blood cell count, BMNC colony cultures, and the proportion of BM CD34^+^ cells than CsA or MSC or T_reg_ cells alone. Simultaneously, the addition of MSC or T_reg_ to CsA could further reduce the inflammatory factors when compared with CsA monotherapy, although a significant improvement in the CD4^+^/CD8^+^ T cell ratio was not observed. Upon comparing the treatment with CsA + UC-MSC and CsA + UCB-T_reg_, we observed that CsA + UC-MSC seemed to recover the blood cell count better than CsA + UCB-T_reg_ on d14 but not on d28. Conversely, the CsA + UCB-T_reg_ group showed a greater reduction in inflammatory factor levels. To the best of our knowledge, this effect has not previously been reported in the literature.

**Figure 7:**
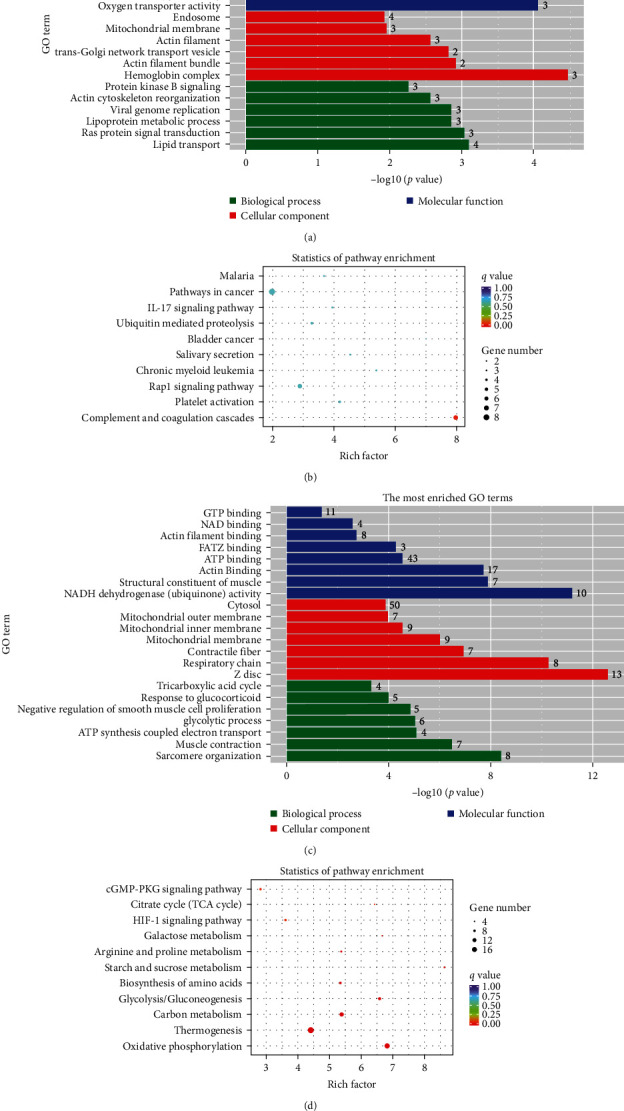
Transcriptome sequence analysis between two kinds of infusion treatments and CsA monotherapy. (a) GO analysis of differential genes between the CsA + UC-MSC and CsA group. (b) KEGG analysis of differential genes between the CsA + UC-MSC and CsA group. (c) GO analysis of differential genes between the CsA + UCB-T_reg_ and CsA group. (d) KEGG analysis of differential genes between CsA+UCB-T_reg_ and CsA group. CsA, cyclosporine A; MSC, mesenchymal stem cells; UC, umbilical cord; UCB, umbilical cord blood; T_reg_, regulatory T cells; GO, gene ontology; and KEGG, Kyoto Encyclopedia of Genes and Genomes.

Transcriptome sequencing of differently treated mice revealed that CsA + UC-MSC treatment could significantly alter histone methylation and the complement and coagulation cascade reaction pathways, whereas the treatment with CsA + UCB-T_reg_ upregulated energy metabolism-related processes, including glycolysis, tricarboxylic acid cycle, and oxidative phosphorylation, compared with CsA alone. Moreover, treatment with CsA + UC-MSC and CsA + UCB-T_reg_ showed differences in the histone methylation process, energy metabolism-related processes, and SOD activity (Figure 7).

Histone methylation refers to the process of altering the interaction between histone and chromatin by adding or removing methyl groups to histone lysine or arginine residues, which could regulate the replication, expression, damage repair, and other functions of chromatin [30, 31]. Histone methyltransferases and demethylases play key roles in the histone methylation process and can be classified into multiple families according to the functional residues and structural characteristics. Downregulation of KMT2a and KMT2c (lysine methyltransferase 2a/2c) and upregulation of KDM4a (lysine demethylase 4a) were detected in the CsA + UC-MSC group, which eventually decreased the histone methylation. Although these genes have not been identified in AA, upregulated expression of KMT2a and KMT2c and downregulated KDM4a expression, compared with normal control, have been reported in patients with autoimmune dermatosis [32]. Agger et al. [33] found that the hematopoietic function was significantly reduced in a KDM4a-gene-knockdown mouse model, while another study reported that hypermethylation of the H3K36 site was related to the enhancement of MSC adipogenic differentiation tendency, which could be suppressed by KDM4a expression [34]. These data confirm that the *KDM4a* gene is crucial for long-term hematopoiesis. Thus, the addition of UC-MSC to CsA could alter histone methylation-related enzyme genes and may affect hematopoiesis and immune regulation. The complement and coagulation cascade pathways are another pathway with significant enrichment of differential expression genes. Single-cell sequencing and proteomic and metabonomic analyses of CD8^+^ T cells from patients with AA have revealed that the complement and coagulation cascade pathway is significantly activated in these patients [35].

The addition of UCB-T_reg_ to CsA significantly enriched genes in the energy metabolism process, primarily related to glycolysis, tricarboxylic acid cycle, and oxidative phosphorylation. Alterations in the energy metabolism mode of hematopoietic stem cells (preferring anaerobic glycolysis or aerobic respiration) may underlie the pathogenesis of several kinds of hemopathy [36, 37, 38], and the unique changes after UCB-T_reg_ addition may be mediated via these pathways.

CsA + UC-MSC significantly enriched the SOD activity-related pathway when compared with CsA + UCB-T_reg_. SOD is an important antioxidant enzyme that can protect cells from reactive oxygen species (ROS)-mediated damage. ROS can cause oxidative damage to hematopoietic stem cells and osteoblasts, increase fat production in BM, and induce hematopoietic failure by directly attacking hematopoietic stem cells and inhibiting the hematopoietic microenvironment. ROS played a central role in the onset of AA in the chemotherapy-induced AA animal model, and the expression of SOD in this animal model was significantly reduced [39, 40]. CsA + UC-MSC significantly upregulated the SOD activity-related pathway, which may further improve the therapeutic effects.

## 5. Conclusions

In summary, the addition of UC-MSC or UCB-T_reg_ to CsA could further improve the efficacy of CsA in treating an AA mouse model. The treatment with CsA + UC-MSC elicited superior hematologic improvements, whereas CsA + UCB-T_reg_ was advantageous in immune regulation. The addition of different cord blood components can function through different pathways. In future investigations, we plan to explore the safety and efficacy of treatment with UCB-derived cells combined with CsA in humanized AA animal models, closely followed by early clinical trials. Collectively, these results suggest the potential for improving the treatment of AA in humans using our approach.

## Figures and Tables

**Table 1:** Treatment regimens for AA mice in different groups.

Group number	Number of mice	Intraperitoneal injection treatment regimen	UC-MSC or UCB-T_reg_ infusion treatment
A1 (CsA + UC-MSC)	9	CsA, 50 mg/(kg·d), days 0–10	UC-MSC, 1 × 10^6^/kg, once
A2 (CsA + UCB-T_reg_)	9	CsA, 50 mg/(kg·d), days 0–10	UCB-T_reg_, 1 × 10^6^/kg, once
B1 (UC-MSC)	9	Equivalent volume of saline (blank control)	UC-MSC, 1 × 10^6^/kg, once
B2 (UCB-T_reg_)	9	Equivalent volume of saline (blank control)	UCB-T_reg_, 1 × 10^6^/kg, once
C1 (CsA)	9	CsA, 50 mg/(kg·d), days 0–10	Equivalent volume of saline (blank control)
C2 (blank control)	9	Equivalent volume of saline (blank control)	Equivalent volume of saline (blank control)
C3 (normal control)	9	Normal control mice without TBI or lymph node cell infusion	

*Note*: CsA, cyclosporine A; UC-MSC, umbilical cord mesenchymal stem cells; and UCB-T_reg_, umbilical cord blood regulatory T cells.

## Data Availability

The data used to support the findings of this study are included within the article.
